# Peptide-siRNA nanotherapeutics in arthritis

**DOI:** 10.18632/oncotarget.4439

**Published:** 2015-06-11

**Authors:** Christine T.N. Pham, Hua Pan, Samuel A. Wickline

**Affiliations:** Department of Medicine, Division of Rheumatology, Washington University School of Medicine, Saint Louis, MO, USA

RNA interference (RNAi) is a process that involves the delivery of small single stranded RNA molecules into mammalian cells, resulting in the sequence-specific cleavage of complementary mRNA and the silencing of specific gene expression. These small RNA molecules called small interfering RNAs (siRNAs) are the focus of intense research due to their potential therapeutic uses in various disease processes ranging from cancer to autoimmune and inflammatory conditions. However, critical barriers to the delivery of siRNAs *in vivo* remain. “Off-target” effects due to the suppression of closely related or unrelated genes might lead to unintended and potentially harmful host responses. Additionally, unprotected siRNAs are highly unstable once introduced into the circulation, with half-life of less than ten minutes, as they are quickly degraded by endogenous nucleases.

Effective delivery of siRNAs to the desired cells/tissues requires specialized delivery strategies as naked siRNAs penetrate cell membrane poorly due to their negative charge. Different carrier systems have been developed to enhance siRNA delivery, including cationic lipid- and polymer-based nanoparticle formulations. However, potential toxicities have hindered the clinical translation of these platforms. Several cell-penetrating peptides (CPP) have also been used to enhance intracellular uptake of siRNAs. Excessive endosomal entrapment, however, remains the primary barrier to many CPP-mediated delivery systems.

Melittin is a well-studied peptide with cell lytic potential through its lipid membrane binding and pore forming property. Our studies of the peptide structure-function relationships and peptide-lipid interactions led to modifications that yielded an amphipathic cationic peptide called “p5” that serves as a “peptide linker” capable of rapidly functionalizing lipid membranes with cargo such as targeting ligands, drugs, and imaging contrast agents but is now substantially less lytic (a 5,700% decrease) compared to native melittin [1-3]. Further modifications of p5, based on studies of nucleotide-peptide interactions, were shown to enable a number of other useful attributes such as protected siRNA transport in circulation and rapid delivery to target tissues through passive permeation in addition to endosomal escape after pinocytotic uptake and concomitant rapid release of siRNA that depends on endosomal acidification and peptide protonation [4, 5]. Unlike the present class of lipid-based carriers, our peptide carrier for siRNA delivery is not sequestered in the reticuloendothelial system but is cleared renally. Avoidance of liver/spleen sequestration and renal clearance of active peptide-siRNA nanoparticles also serve to promote more focused tissue interactions, particularly under conditions of inflammation and enhanced vascular permeability as in cancers [5]. Finally, the approach is agnostic as to the exact oligonucleotide sequence, and the entire complex can be formed within as little as ten minutes by a simple mixing procedure that allows noncovalent, self-assembly of cationic and anionic moieties into a ~55 nm nanocomplex ready for direct injection or else fully stabilized for later use by mixing in albumin. The resulting material is stable in both blood and plasma from hours to days as previously reported [4, 5].

Rheumatoid arthritis (RA) is a chronic inflammatory condition affecting approximately 1% of the population worldwide. Despite recent advances in therapeutics, 30-40% of RA patients fail to respond to these new medications or are forced to cease treatment due to adverse effects. In our recent work [6], we examined the ability of our peptide-siRNA nanoplatform to silence p65, a major effector of the NF-κB canonical signaling pathway that controls the expression of various inflammatory mediators, including TNF-α, IL-1β, and IL-6. Although NF-κB represents an attractive therapeutic target for inflammatory arthritis, its generalized suppression can result in serious host toxicity, as NF-κB is critically involved in innate and adaptive immune responses. Using the anti-collagen antibody induced arthritis (CAIA) model we showed that anti-p65 siRNA nanocomplexes homed to the inflamed paws through leaky vasculature, localized to synovial macrophages and suppressed ongoing arthritis in 6-8 week-old male DBA1/J mice while equivalent doses of naked anti-p65 siRNA did not alter the disease progression. Moreover, joint-associated levels of multiple inflammatory cytokines (TNF-α, IL-1β, IL-6, and MCP-1) were all profoundly suppressed in mice treated with anti-p65 siRNA nanocomplexes. We showed that p65 mRNA and protein expression were specifically downregulated only in the paws of these animals while levels of related NF-κB family members (p100/52, p105/50, RelB) were preserved. In addition, p65 and related NF-κB protein levels in off-target organs such as liver, spleen, brain, and kidneys were unperturbed. More importantly, our initial studies suggest that serial systemic injections of the peptide-siRNA nanocomplexes did not elicit complement activation or specific humoral response [6]. In summary, modifications of the native melittin led to the formulation of a self-assembling nanoplatform that may realistically offer safe, effective and highly specific *in vivo* delivery of siRNAs for the treatment of RA and potentially many other inflammatory conditions that require chronic and repeated injections.
